# Synaptic weighting in single flux quantum neuromorphic computing

**DOI:** 10.1038/s41598-020-57892-0

**Published:** 2020-01-22

**Authors:** M. L. Schneider, C. A. Donnelly, I. W. Haygood, A. Wynn, S. E. Russek, M. A. Castellanos-Beltran, P. D. Dresselhaus, P. F. Hopkins, M. R. Pufall, W. H. Rippard

**Affiliations:** 1 National Institute of Standards and Technology, Boulder, CO 80305 USA; 2 Stanford University, Department of Electrical Engineering, Stanford, CA 94305 USA; 3 Lincoln Laboratory, Massachusetts Institute of Technology, Lexington, MA 02420 USA

**Keywords:** Superconducting devices, Electrical and electronic engineering, Applied physics

## Abstract

Josephson junctions act as a natural spiking neuron-like device for neuromorphic computing. By leveraging the advances recently demonstrated in digital single flux quantum (SFQ) circuits and using recently demonstrated magnetic Josephson junction (MJJ) synaptic circuits, there is potential to make rapid progress in SFQ-based neuromorphic computing. Here we demonstrate the basic functionality of a synaptic circuit design that takes advantage of the adjustable critical current demonstrated in MJJs and implement a synaptic weighting element. The devices were fabricated with a restively shunted Nb/AlO_x_-Al/Nb process that did not include MJJs. Instead, the MJJ functionality was tested by making multiple circuits and varying the critical current, but not the external shunt resistance, of the oxide Josephson junction that represents the MJJ. Experimental measurements and simulations of the fabricated circuits are in good agreement.

## Introduction

Software based deep neural networks have proven to be extremely useful for many classes of problems in recent years^1–4^. However, the time that it takes to train modern large scale networks is one of their key limiting factors. Hardware acceleration of the training is largely utilized with common adoption of graphics processing units (GPUs)^5^, and the design and implementation of application-specific integrated circuits (ASICs) such as tensor processing units (TPUs)^6^. However, these accelerators for deep learning still suffer from the von Neumann memory-logic bottleneck and are largely limited by the memory access time needed to store and retrieve very large matrices of synaptic weights^7^. This in part has led to a renewed exploration of neuromorphic hardware with the goal of further accelerating the advance of artificial neural networks. One promising hardware platform can be made from naturally spiking Josephson junctions (JJs)^8–12^. Because of this spiking behavior, JJs have been proposed to simulate the interactions among neurons, and have demonstrated biologically realistic neuron behavior with only two junctions^13^. Further, they have been proposed as a way to implement stochastic neural networks^14,15^, and have been demonstrated to implement a sigmoid like transfer function of fast voltage spikes^10^.

The recent demonstration of a magnetic Josephson junction (MJJ) having a critical current that can be tuned in an analog fashion has reduced the number of elements required to implement high speed (>10 GHz), energy efficient (<1 aJ/spike) artificial neural networks^16,17^. Simulations have shown that a network of JJs with fixed critical currents *I*_*c*_ and MJJs with variable *I*_*c*_ could be used to mimic the basic neuron synapse behavior of feed-forward neural networks^18^. The concept for the circuit functionality is to pass a varying amount of an single flux quantum (SFQ) spike into an output superconducting quantum interference device (SQUID) depending on the critical current *I*_*c*_ of the MJJ, which is acting as the synapse. The remainder of this input SFQ pulse would be shunted to ground through a fixed inductor. In this scheme, there would then be several synaptic elements coupled into a single output SQUID, which acts as the post-synaptic threshold-neuron. The output SQUID would fire a pulse further into the network once it’s threshold is crossed.

## Results

Here we demonstrate a quasi-static proof-of-principle for the synaptic weighting and post-synaptic threshold-neuron behavior experimentally. The circuits were fabricated with the Lincoln Laboratory SFQ5ee process. The JJs were superconductor-insulator-superconductor (SIS) Nb/AlO_x_-Al/Nb junctions, with critical current density *J*_*c*_ of 100 µA/µm^2^ and had a minimum diameter of 700 nm. More details about the process can be found in ref. ^19^. Since these circuits rely on fixed *I*_*c*_ JJs, we emulate the variable *I*_*c*_ of the proposed MJJ synapse by fabricating four nearly identical circuits where the only change is the *I*_*c*_ of the JJ in the synaptic position. The quasi-static input current is fed into the synaptic part of the circuit. The amount of this input current that is coupled into the output SQUID can then be measured as the reduction in the critical current of the output SQUID circuit.

While reprogrammable synapses implemented with MJJs will offer the most flexibility in a neuromorphic hardware design, fixed weight synapses, such as those implemented here, can perform inference in a purpose-built hardware neural network. In this type of implementation, one could determine the weights of the target neural network with the usual algorithms, e.g. back propagation. These weights would then be mapped to the superconducting critical current of the synaptic elements. Such a hardware neural network could offer significant speed enhancement compared to the equivalent inference run by a software neural network. This type of inference could also be viewed as high speed hardware signal filters and might have applications in communications or radar signal processing, where superconducting analog to digital converters have already demonstrated noise improvements compared to room temperature technologies^20^.

**Figure 1:**
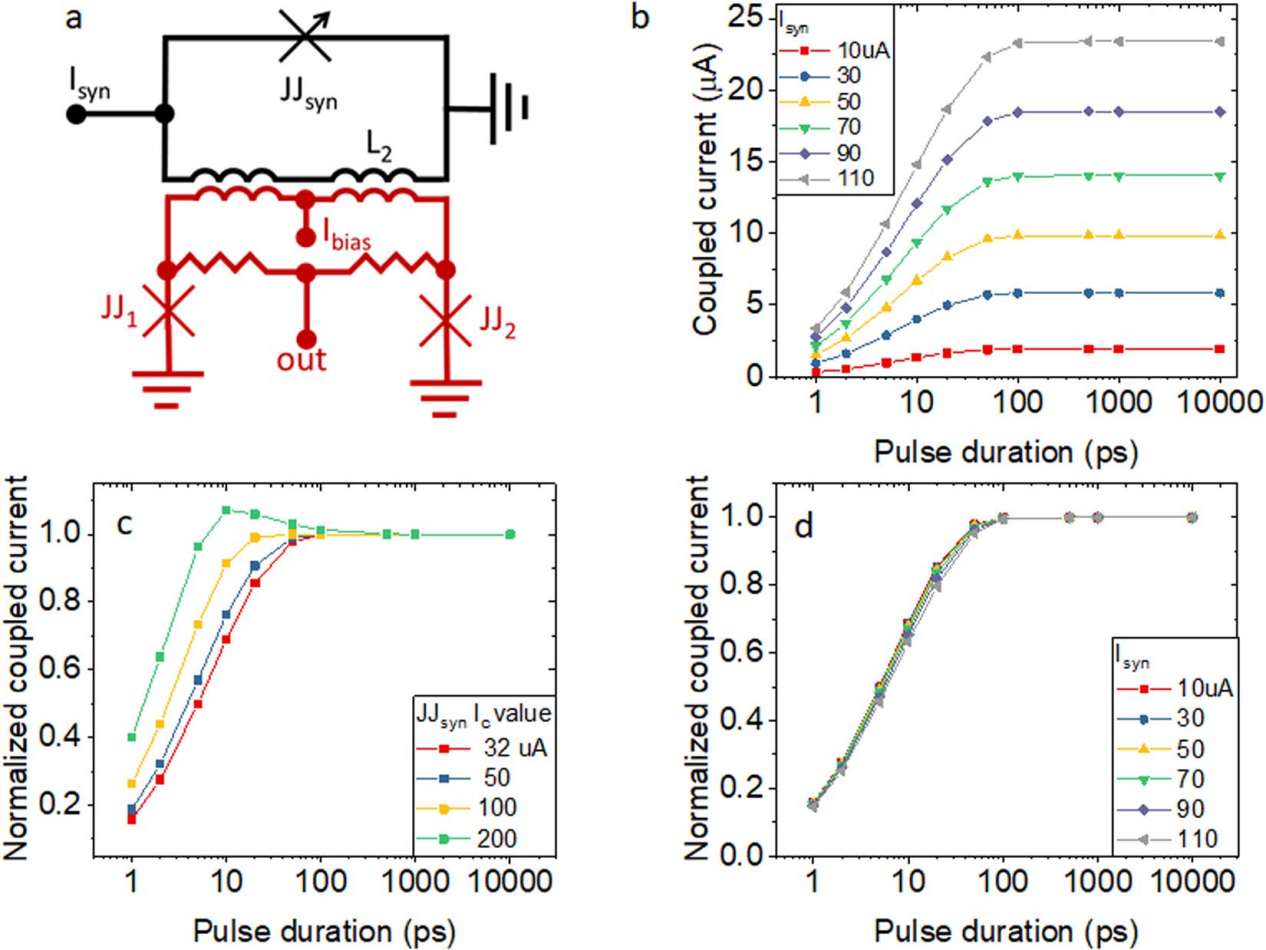
Simulations of the behavior of the JJ synapse. **(a)** Schematic circuit diagram that is being tested with the synaptic portion shown in black and the output SQUID portion shown in red. **(b)** Simulation results of the current coupled into the output SQUID of the 32 µA synapse, as a function of pulse duration, for pulses applied at the input *I*_*syn*_ with varying pulse amplitudes. **(c)** Simulation results of the normalized current coupled into the output SQUID as a function of pulse duration for different values of Ic, the coupled current is normalized to the value measured at 10 ns for each of the *I*_*c*_ values. **(d)** Simulation results of the normalized current coupled of the 32 µA *JJ*_*syn*_ in to the output SQUID versus pulse duration for various input pulse amplitudes.

Figure 1a shows the basic circuit diagram for the synaptic element (black) and the accompanying readout SQUID (red). The presynaptic neuron would input a signal to *I*_*syn*_ and the postsynaptic neuron would fire a signal at out. In a network of neuronal spiking JJs and synaptic elements, the signal out in Fig. 1a would be connected to a subsequent neuron, which would provide the return path. The current design utilized bias resistors for ease of testing. The input current to the synaptic element is a quasi-static bias current in the experiments. Simulations were run as a function of time to confirm that the measured quasi-static behavior agrees with simulation results and to understand how this will relate to the envisioned pulsed SFQ operation in future designs. Figure 1b shows simulation results that indicate that the circuit design will work with pulsed operation. It should be noted that, as intended, no spiking of the synaptic JJ was observed with any of the pulses up to 110 µA.

The synaptic weighting function is accomplished by varying the amount of the input current that is coupled into the output SQUID. The amount of current that is coupled into the output SQUID is determined by the inductive splitting between the synaptic JJ (*JJ*_*syn*_) and the fixed coupling inductor. This splitting can be adjusted by varying the *I*_*c*_ of *JJ*_*syn*_ and therefore the Josephson inductance of *JJ*_*syn*_, which is given by^21^

1 $$L=\frac{{\Phi }_{0}}{2\pi {I}_{c}}\frac{si{n}^{-1}(i/{I}_{c})}{i/{I}_{c}},$$

where *i* is the current through the junction, and Φ_0_ = 2.07 × 10^−15^*V*·*s* is the single flux quantum. The value of the Josephson inductance varies from ${L}_{1}={\Phi }_{0}/2\pi {I}_{c}$ when $i=0$ to $(\pi /2){L}_{1}$ when $i={I}_{c}$.

The synaptic circuit operates based on the dependence of the Josephson inductance on *I*_*c*_ of *JJ*_*syn*_. Because the inductance is inversely proportional to the critical current, smaller *I*_*c*_ junctions will have a higher inductance value. In addition, when the current passing through the junction is increased to near the critical current level, the inductance of the junction will increase slightly. When used in the synaptic circuit element shown in Fig. 1a, low *I*_*c*_ junctions will couple more of the incoming current into the output SQUID. If the incoming current is fixed in value, this means that the lower *I*_*c*_ junctions will be operating closer to their *I*_*c*_ value. In this case the inductance of the JJ will be increased, leading to a stronger coupling into the output SQUID, which reinforces the desired behavior of the synaptic element.

The junctions in our circuits have been externally shunted (not shown in the circuit diagram for readability) with a 0.33 Ω shunt resistor. This shunt resistor has a much lower resistance value than the JJ barrier and therefore is also roughly the normal state resistance (*Rn*). While a higher value of *Rn* could have been chosen to achieve higher characteristic frequencies and lower damping, it was desired to fabricate circuits with *Rn* comparable to that demonstrated in previous MJJs, since these are ultimately the target synaptic junctions.

To better understand the impact of the input pulse duration, time domain simulations in WRSPICE^22^ were performed. Figure 1b shows the simulated current coupled into the output SQUID for a circuit where *JJ*_*syn*_ has *Ic* = 32 µA. A pulse was applied to the input marked on the circuit diagram as *I*_*syn*_. The coupled current was sampled at the mutually coupled inductor in the output SQUID with *I*_*bias*_ = 0. The expected coupled current for the quasi-static case is given by the inductive divider, which can be approximated as ${I}_{coupled}={I}_{syn}({L}_{1}/({L}_{1}+{L}_{syn}))\ast k,$ where k is the coupling constant of the inductors, *L1* is the fixed inductor which is mutually coupled to the output SQUID, and *L*_*syn*_ is the inductance of the synaptic JJ. The values from this approximation agree well with the long pulse duration results in Fig. 1b. However, there is a roll-off in inductive splitting for shorter pulse durations in the simulations. The roll-off is smooth with respect to pulse duration and therefore exceeding the speed of the junctions should not cause catastrophic circuit failure, but rather a reduction in the dynamic range of the splitting. The simulation results show that pulse durations >100 ps yield the full dynamic range of the synaptic circuit, which is consistent with a roughly 3 GHz operating speed.

The synaptic circuits are envisioned to work with MJJs in the future. In this mode of operation, the *I*_*c*_ of *JJ*_*syn*_ can be adjusted without changing the resistance of the junction. Figure 1c shows the simulated value of the current coupled into the output SQUID for 4 different *I*_*c*_ values of *JJ*_*syn*_ as a function of pulse duration. We see that the roll-off at shorter pulses is similar for the different *I*_*c*_ values, as is desired. The input current to *I*_*syn*_ had an amplitude of 10 µA for these simulations. The peak current value coupled into the output SQUID was normalized to that of the value for the longest pulse duration. In all cases simulation results showed no significant difference in the value of the split current between 1 ns and 10 ns, indicating that this region is near the quasi-static limit where the fabricated circuits where tested as described below. The slight increase in the peak coupled current for the 200 µA junction circuit around 10 ps correlates with the slight decrease in damping for those junctions. Please see appendix A for further discussion of the peak in current transfer at 10 ps. With the exception of this peak, all of the circuits show a similar reduction of the coupled current for short pulses, indicating a reduction in the dynamic range of the circuit for short pulses. The reduction in the dynamic range of the circuit is a result of the reduction in the maximum current coupled into the output SQUID. However, since the pulse duration in an SFQ circuit is known, the output SQUID can be designed to operate with the lower maximum current coupling mitigating any dynamic range issues that result from a reduced current value for short pulses.

Ideally the synaptic circuits would be able to operate with a wide pulse amplitude range. Figure 1d shows simulations of the effect of changing pulse amplitude normalized to the maximum couple current. The data are the current coupled into the output SQUID for a 32 µA *I*_*c*_ synapse junction, which had the strongest reduction in dynamic range as seen in Fig. 1c. The maximum input current was chosen to bias *JJ*_*syn*_ near *Ic*, in this case an input current of 110 µA, which lead to a peak current through *JJ*_*syn*_ of 31 µA. In the plot of Fig. 1d, the coupled current was normalized to the 10 ns coupled current value for easy comparison. These data show that the circuit is largely insensitive to input current amplitude up to 110 µA, though there is a slight reduction of the coupled current at high input currents amplitudes. This insensitivity to input current amplitude will make future circuit design much more forgiving.

**Figure 2:**
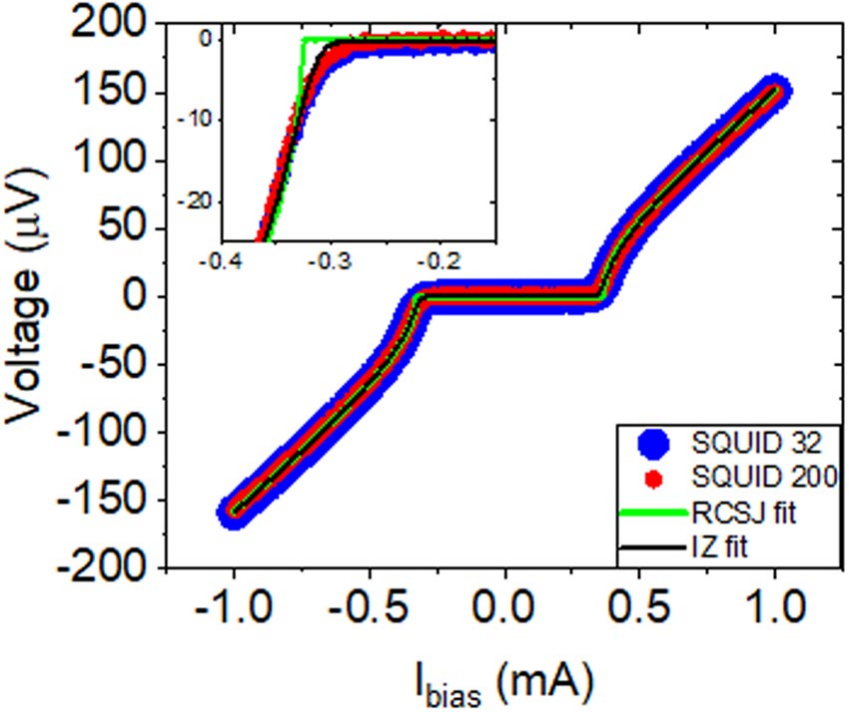
Voltage versus current taken on the output SQUID for the *JJ*_*syn*_ with *Ic* = 32 µA circuit in blue and *JJ*_*syn*_ = 200 µA circuit in red. The *Ic* = 32 µA and *Ic* = 200 µA data are in good agreement as expected. Fits to the RSJ model and IZ model are shown in green and black, respectively. Inset is a zoom in of the negative knee.

The behavior of the four circuits types was experimentally measured by fitting quasi-static Vout vs. *I*_*bias*_ curves of the output SQUID while varying the input current at *I*_*syn*_. Representative voltage versus current data for the output SQUID with zero applied current at *I*_*syn*_ is shown in Fig. 2 for both the circuit with a 32 µA and a 200 µA synaptic junction. As expected, without a bias applied through *I*_*syn*_ there is very little difference between the circuits indicating good fabrication uniformity. The fit to the resistively shunted junction (RSJ) model, shown in yellow in Fig. 2, does not capture the rounding near the critical current. We choose this model because at 70 fF per 100 µA, the effect of the capacitance is negligible. These circuits were all made with over damped JJ’s with a McCumber Parameter between 0.0002 at 32 µA and 0.01 at 200 µA. The plasma frequency for these junctions is around 2 THz but the circuit frequency would be limited by the L/R time constant which was as low as 30 GHz for the 32 µA JJ circuit.

The rounding effect that is not captured by the RSJ model is particularly obvious at low critical current values, as has previously been reported^23^. A more accurate fit can be made if the effect of noise fluctuations from the attached electronics is considered such as the form from Ivanchencko and Zil’berman (IZ)^24,25^.

2 $$V={I}_{c}{R}_{n}(\frac{I}{{I}_{c}}-{ {\mathcal I} }_{-}+{ {\mathcal I} }_{+}),\,I\ge 0$$

where ${ {\mathcal I} }_{\pm }=\frac{{ {\mathcal I} }_{1\pm i\gamma }({\gamma }_{c})}{2i{ {\mathcal I} }_{\pm i\gamma }({\gamma }_{c})}$, $\gamma =\frac{I\hslash }{2e{k}_{B}{T}_{eff}}$, and ${\gamma }_{c}=\frac{{I}_{c}\hslash }{2e{k}_{B}{T}_{eff}}$. Where ${ {\mathcal I} }_{\nu }(z)$ is a modified Bessel function of the first kind, *T*_*eff*_ is the effective noise temperature in the measurement system, e is the elementary charge, and *k*_*B*_ is the Boltzmann constant. Fitting results for the four different circuits are listed in Table 1.

**Table 1:** Table of fit parameters obtained with the IZ method at zero applied *I*_*syn*_ bias.

*Ic* value of *JJ*_*syn*_	*Ic* (µA)	*Rn* (Ω)	*T*_*eff*_ (K)
32 µA	353 ± 1 µA	163.1 ± 0.1 mΩ	144 ± 2 K
50 µA	351 ± 1 µA	163.4 ± 0.1 mΩ	120 ± 2 K
100 µA	362 ± 1 µA	163.2 ± 0.1 mΩ	159 ± 2 K
200 µA	352 ± 1 µA	164.0 ± 0.1 mΩ	136 ± 2 K

Note that the measured *Rn* values are expected to be half the shunt resistance value because there are two JJs in parallel in the output SQUID. We would have expected the noise temperature to be consistent between the various circuits since they were measured with the same experimental setup. However, the fits show variations that are likely a result of the subtle differences in the measurement circuits, for example the pads were wire-bonded from the chip to a carrier board which was connected to a dunk probe. The wire bond connections could vary as could any noise pickup between the different wires leading out of the dunk probe. The critical current uniformity of ≈ 3% looks very promising for circuit margins, though it is from a very small sample. Similarly, the small variation in the normal state resistance <1% demonstrates process control that should enable larger scale neuromorphic SFQ circuits.

**Figure 3:**
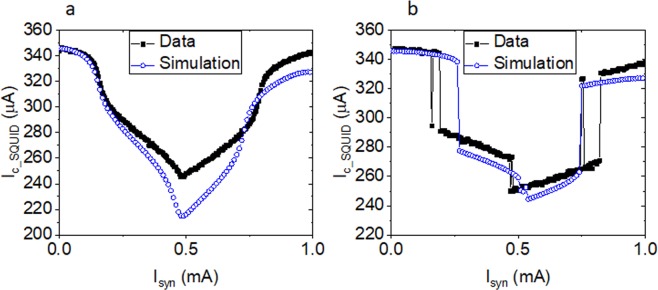
**(a)** Experimental data (black squares) and simulations (open blue circles) of the *I*_*c*_ of the SQUID as a function of bias applied as *I*_*syn*_ for the circuit with *JJ*_*syn*_ *Ic* = 32 µA. **(b)** Experimental data (black squares) and simulations (open blue circles) of the *I*_*c*_ of the SQUID as a function of bias applied as *I*_*syn*_ for the circuit with *JJ*_*syn*_ *Ic* = 200 µA.

Figure 3 shows the data from fabricated circuits and outputs from simulations for *I*_*c*_ of the output SQUID as a function of the bias applied at *I*_*syn*_. Data are shown in closed black squares for the circuits with synaptic critical current values of 32 µA and 200 µA in Figs. 3a,b respectively. Outputs from simulations are shown in the same figures in blue open circles. These curves show the broad range of change to the output SQUID critical current that is available depending on the incoming current into the synapse. More importantly, the difference between 32 µA and 200 µA synaptic JJ value circuits shows the weighting change that is available with this change in *Ic*.

While there is good qualitative agreement between the data and the simulations shown in Fig. 3, several details are not captured. Parasitic inductances are not considered in the simulations, which likely explains some of the differences, such as the depth of the modulation. To find better agreement with the period of oscillation, the inductor values in the SQUID loops in the simulations, which are nominally the same in the physical circuits, needed to be adjusted from 3.5 pH for the 32 µA circuit to 4.2 pH for the 200 µA circuit. This ≈ 15% change in value is not consistent with the variation measured in other circuit parameters. In order to better understand the ability of the simulations to predict the behavior of the tested circuits, the inductor values are fixed at 3.5 pH, as measured by independent test structures, for all other simulations.

Anomalies in the data can be seen in a few spurious *I*_*c*_ values in Fig. 3b. Considering the synaptic part of the circuit (black Fig. 1a) as an RF SQUID, the screening parameter is given by ${\beta }_{L\_RF}=2\pi L{I}_{c}/{\Phi }_{0}$^21^. The circuit in Fig. 3b has the largest *β*_*L*_ with a value of about 4.5, which allows for hysteretic flux behavior. Noise in the current source or thermal activation when highly biased during the measurement could have led to unintentional switching between the two stable flux states of the RF-SQUID loop with JJ_syn_ in this case. This is an important consideration for future synapse design as unintentional switching would lead to errors in the circuit. While the data do not seem to point at thermal activation causing these fluctuations and the thermal activation of the smallest junction (32 µA) is less than 10^−17^ when biased at 70% of *Ic*. Thermal activation cannot be ruled out because of the high bias currents used in these experiments. To minimize this effect, future synaptic design should maintain βL < 1. Because of the potential for errors and the large steps in the output *I*_*c*_ values, the range of currents applied to *I*_*syn*_ was limited below the jumps to < 150 µA for the rest of the analysis of these circuits.

**Figure 4:**
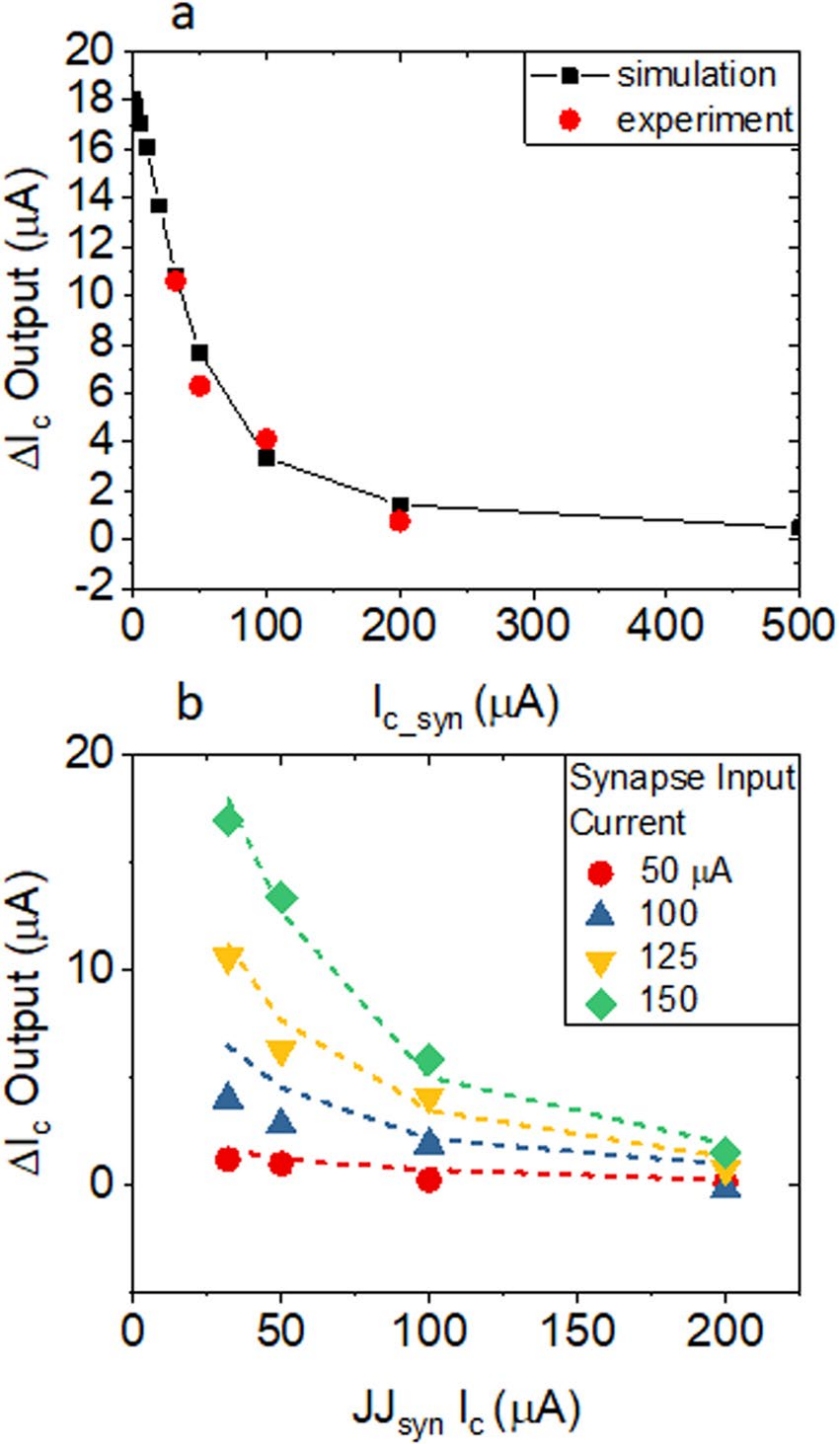
**(a)** Change of the *I*_*c*_ of the SQUID as a function the critical current value of *JJ*_*syn*_ with *I*_*syn*_ = 125 µA. Simulated values are shown in black squares and measured values from the four circuits fabricated here are shown in red circles. **(b)** Change of the *I*_*c*_ of the SQUID as a function the critical current value of *JJ*_*syn*_ for varying values of *I*_*syn*_. Simulation results are shown as dashed lines and data points with symbols defined in the legend.

In the the operating region of *I*_*syn*_ < 150 µA, the output SQUID behavior (the result of the synaptic transfer function) is a function of both *I*_*c*_ of *JJ*_*syn*_ and *I*_*syn*_ bias, which represents the operating region of the neuromorphic circuit. The quantity of interest is the change in the *I*_*c*_ value of the output SQUID. This is measured as the zero *I*_*syn*_ value of the output SQUID minus the *I*_*syn*_ biased value of the output SQUID *Ic*, which we denote as *ΔIc*. Figure 4a shows *ΔIc* as a function of the synapse *I*_*c*_ when *I*_*syn*_ is biased at a constant 125 µA. The simulation results over a broad range of *I*_*c*_ values is shown in black and the data from the four circuits tested with *I*_*c*_ values between 32 µA and 200 µA is shown in red.

Figure 4b compares *ΔIc* of the output SQUID as a function of the *JJ*_*syn*_ *I*_*c*_ for a range of different *I*_*syn*_ bias values. The dashed lines are results from simulation where all parameters are held fixed except the *I*_*c*_ values of the synaptic JJ and the bias values are denoted. The large symbols are the experimentally measured values from the four circuits. There is reasonable agreement between simulations and experiments and importantly both simulated and measured circuits behave monotonically as the *I*_*syn*_ bias value is increased. This implies that a precise input pulse amplitude is not required for future circuits designs and that parameters such as fan-in and fan-out can be adjusted with circuit design with a nominally smooth behavior in the synaptic function. While a larger neuromorphic circuit architecture will need to be worked out in detail, the agreement between experiment and simulations and the generally smooth response of the synaptic circuit are very promising.

While the general response in Fig. 4b is promising, there are deviations between the simulated and measured response particularly for the 100 µA JJ_syn_ circuit. If we allow parameters to vary in the simulations, then we can force better agreement between the simulation and measurement. This discrepancy points to the limitation of simple simulations to predict fabricated circuit behavior. There are two important takeaways for future neuromorphic circuit design. One is that simulations should vary parameters within known process variations to predict the variation in behavior of individual elements, e.g. *I*_*c*_ values and inductor values. The fine tuning of the synaptic weight values in an adjustable hardware technology should be able to compensate for these process variations. However, on a large scale, such fine tuning will likely be overly cumbersome. Thus, for neuromorphic SFQ circuit designs, which are inherently analog, a self-learning behavior of the analog/synaptic portions of the circuits will be very advantageous, if not required.

## Discussion

While the experiments and simulations here are all single synapse tests, it is also important to note that multiple synapses would be used in any computational network. We believe that multiple synapses can feed a single loop coupled to the output SQUID neuron. More detail about such a system of multiple synapses feeding a post synaptic SQUID neuron and simulations of a 9 pixel classifier can be found in ref. ^17^. It is worth exploring a few key attributes that will be necessary to implement such a system with magnetic Josephson junctions. First, the magnetic nanoclusters need to be stable above the critical temperature of the superconducting material, *e.g*. Nb. In this case if one needs to use a global magnetic field to set the weights of the synapses, then a defluxing operation where the temperature of the circuit is raised above the superconducting critical temperature can be performed after the weight training, without effecting the weights. In addition, the cluster orientation in this scheme should not be effected by the single flux quantum pulses emitted by the SQUID neurons.

We briefly discuss the potential for a JJ/MJJ neural network as compared to modern CMOS. JJ/MJJ based neural networks can leverage the development of digital JJ circuits, which are near 106 JJ’s per square cm^26,27^. While these densities are no-where near modern CMOS the JJ fabrication process continues to scale and at roughly 106 JJ’s per cm2 some smaller scale applications may be within reach. The first potential advantage of JJ/MJJ neural networks lies in the speed at which they could operate. JJ based circuits have been demonstrated at speeds greater than 700 GHz^28^. While the circuits demonstrated here are highly overdamped and would be limited to speeds closer to 30 GHz, these speeds are still quite favorable compared to modern CMOS CPUs/GPUs or specialized neural network ASICS, which typically operate below 6 GHz. In addition to the speed of the JJs, because the SFQ pulses are transmitted along superconducting wires, there are no associated RC time constants and the speed of SFQ pulse propagation is roughly 1/3 the speed of light in vacuum. Energy efficiency of a small-scale circuit would be dominated by cooling overhead. However, if the circuits prove to be powerful enough to be scaled to a larger system then they could also be more efficient that modern CMOS circuits^29^. It is also worth noting that the energy required to reorient the magnetic clusters in the MJJ synapses has been demonstrated to be as low as 3 aJ^16^, which again compares favorably to modern CMOS. These potential advantages in speed and power are compelling reasons to further investigate this potential technology. However, because the demonstrations to date have been at the single device level, there is much more work that will need to be done to fully understand how a large-scale JJ/MJJ neural system will compare to modern CMOS.

In conclusion, we have designed and fabricated four independent JJ circuits that test the functionality of JJ synapses. In these circuits, the Josephson inductance was changed by adjusting the *I*_*c*_ value of the synaptic element. The change in inductance results in the ability to vary the amount of current coupled into an output SQUID. This functionality can be used in neuromorphic SFQ circuits to weight incoming pulses. We show an operating range that smoothly varies between 0 µA and 150 µA. Extension of the range of *I*_*c*_ values in WRSPICE simulations confirms the weighting of the synaptic JJs and agreement with the four circuits that were fabricated. We have previously shown in simulations that software-based feed-forward neural networks can be directly implemented in SFQ circuits; this work provides further simulation and experimental verification of the synaptic weighting elements that compose such circuits.

## Supplementary information

Supplementary information.
