# Supplementary material for: Synaptic weighting in single flux quantum neuromorphic computing
Source: Sci Rep. 2020 Jan 22;10:934. doi: 10.1038/s41598-020-57892-0 (PMC6976664; doi:10.1038/s41598-020-57892-0)
Supplement: Supplementary file 1 — Supplementary information. [file 41598_2020_57892_MOESM1_ESM.pdf]

Supplementary Information

# Synaptic weighting in single flux quantum neuromorphic computing

M. L. Schneider[1*], C. A. Donnelly[1,2], I. W. Haygood[1], A. Wynn[3], S. E. Russek[1], M. A. Castellanos-Beltran[1], P. D. Dresselhaus[1], P. F. Hopkins[1], M. R. Pufall[1], W. H. Rippard[1]

1) National Institute of Standards and Technology, Boulder, CO 80305, USA
2) Stanford University, Department of Electrical Engineering Stanford, CA, 94305, USA
3) Lincoln Laboratory, Massachusetts Institute of Technology, Lexington, MA 02420, USA

## Details of timing in the synaptic signal modulation circuit

The synaptic signal modulation is envisioned to operate at high speeds with SFQ pulses. As such, it is important to understand the timing conditions of the circuit as a function of the $I_c$ value of the $JJ_{syn}$. Such timing considerations can be seen in fig 1c of the main text for the $JJ_{syn}$ with $I_c$ = 200 µA, where a peak occurs in the coupled current around 10 ps. This peak is in part a reflection of how the data were normalized. Figure S1a shows the coupled current as a function of time for a 100 ps input pulse. The current value for normalization was taken at the middle of the pulse time. Thus the 10 ps center pulse time current value was ~ 7 % higher than the 10 ns center pulse time current value, though they had the same peak value. Figure S1b shows the same trace for the simulation of the $JJ_{syn}$ $I_c$ = 100 µA. Note that in this case there is no overshoot in the time trace and therefore no peak in the corresponding data in fig 1c of the main text.

When changing $I_c$ of the $JJ_{syn}$, we also change the screening current, characteristic time constant, and damping parameter. Below we test each of these changes to see which effect dominates the short time scale response in our circuit. Figure S1c shows the time trace with the $\beta_L$ of $JJ_{syn}$ $I_c$ = 200 µA adjusted to match that of $JJ_{syn}$ $I_c$ = 100 µA in the original simulations. Note, that the screening current enhances the short time scale peak, and therefore does not explain the effect.

The slowest characteristic time for the overdamped $JJ_{syn}$ is given by

$$\tau_{RL} = \frac{\Phi_0}{2\pi I_c R}, \qquad (s1)$$

which is about 20 ps for the 200 µA $JJ_{syn.}$ However, if the characteristic time were the dominate effect, we would expect the 100 µA $JJ_{syn}$ to have an even larger effect at 10 ps, since its $\tau_{RL}$ is 10 ps.

Correspondence and requests should be addressed to M.L.S. (email: michael.schneider@nist.gov)

Finally, we are left with the Stewart-McCumber parameter[1,2] $\beta_c$, which represents the damping in the junction with $\beta_c < 1$ being in the overdamped regime, $\beta_c = 1$ being critically damped and $\beta_c > 1$ being underdamped, where

$$\beta_c = \left(\frac{2\pi}{\Phi_0}\right) I_c R_n^2 C \qquad (s2)$$

where $C$ is the junction capacitance. $\beta_c$ for the $JJ_{syn}$ values tested here was between $6.7\times10^{-5}$ and $2.6\times10^{-3}$ corresponding to highly overdamped junctions in all cases. In fig S1d we adjust the shunt resistor in the simulation to increase the damping of the $JJ_{syn}$ with $I_c$ = 200 μA again to match that of the case where $JJ_{syn}$ has $I_c$ = 100 μA. In this case, we see that the short time scale peak is eliminated implying that change in damping caused the short time scale overshoot in coupled current for the $JJ_{syn}$ with $I_c$ = 200 μA.

It is worth noting that a large $I_c$ value $JJ_{syn}$ is intended to couple the least amount of current into the output SQUID. As such, the slightly higher peak current could reduce the dynamic range of the signal modulation if the output SQUID circuit is fast enough to respond to the shot time scale current. In future design considerations, it is important to take this into account by designing the circuit to have consistent dynamics across the intended range of $I_c$ values that a $JJ_{syn}$ would potentially access. For example, if the damping was increased, then all the $I_c$ values could be damped out. If a faster circuit is desired, then the damping would need to be decreased enough for all the $I_c$ values to have a fast response. While, either of these cases would lead to the largest possible dynamic range of coupled current, a slight reduction, e.g. 7 %, should still yield synaptic signal modulation that is quite acceptable.

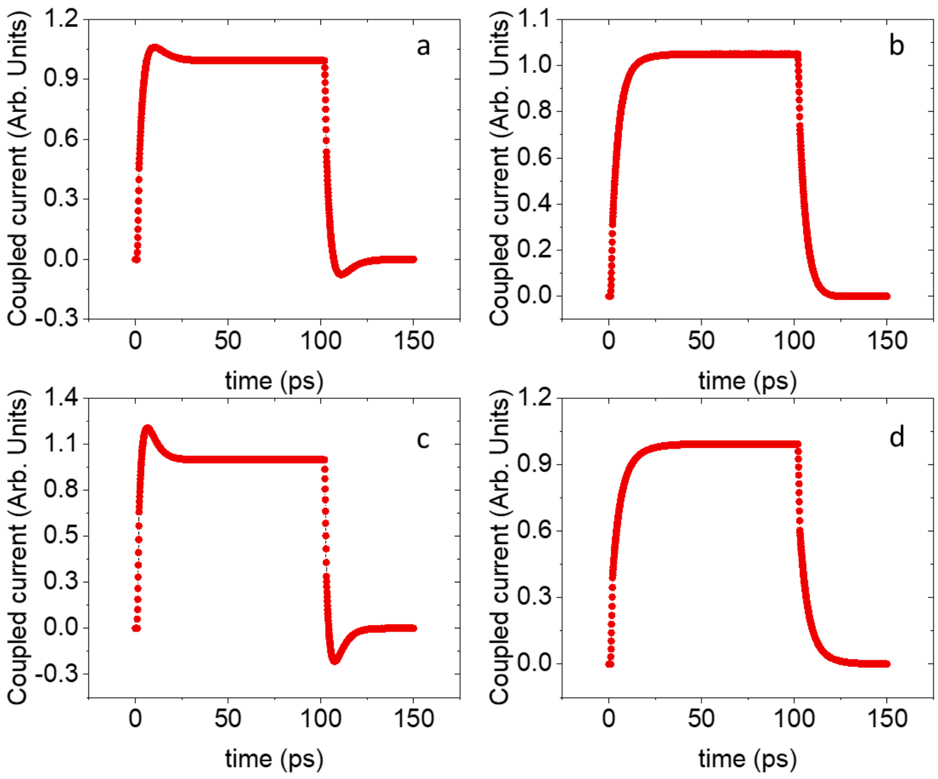

Figure S1. (color online) Coupled current normalized to the value at 50 ps. (a) Time trace of the coupled current with the simulation conditions of fig. 1c of the main text (green), $JJ_{syn}$ $I_c$ = 200 μA, $R_{shunt}$ = 0.33 ohm, and $L_1 = L_2$ = 3.7 pH. (b) Time trace of the coupled current with the simulation conditions of fig. 1c of the main text (yellow), $JJ_{syn}$ $I_c$ = 100 μA, $R_{shunt}$ = 0.33 ohm, and $L_1 = L_2$ = 3.7 pH. (c) Time trace of the coupled current with $\beta_{L\_RF}$ reduced to the same value as the $JJ_{syn}$ = 100 μA case, $JJ_{syn}$ $I_c$ = 200 μA, $R_{shunt}$ = 0.33 ohm, and $L_1 = L_2$ = 1.85 pH. (d) Time trace of the coupled current with $\beta_c$ reduced to the same value as the $JJ_{syn}$ = 100 μA case, $JJ_{syn}$ $I_c$ = 200 μA, $R_{shunt}$ = 0.165 ohm, and $L_1 = L_2$ = 3.7 pH.

**Schmatic layout of the test circuits:**

The circuits were designed for ease of testing and were not optimized for minimizing the space. As such the circuits are rather large, with the largest amount of space being taken up by the inductors in the circuit. A wire width of 2.5 μm was used for the inductors, and that could easily be reduced to sub-micron width. If one works with the narrower width inductor, it will have a higher inductance per micron, which will lead to a more compact layout. The smallest junction that we tested was roughly 0.7 μm in diameter. This was the 32 μA synaptic junction and we did not observe any issues with yeild at this device size, though we only tested three chips. If we were to redesign the circuit to minimize the area, our nominal 7 pH inductor would need to be be about 7.8 μm in total length assume that we designed the wire width to have 0.9 pH/μm. The junctions would remain the same size, which is around 0.7-1.5 μm in diameter depending on the desired $I_c$. Below we show both the schematic layout of the test circuits (fig. S2), and an optical image of one of the actual circuits tested. Both the schematic layout and optical image are for the circuit that has a 100 μA synaptic junction. The SQUID neurons in all circuits are the same.

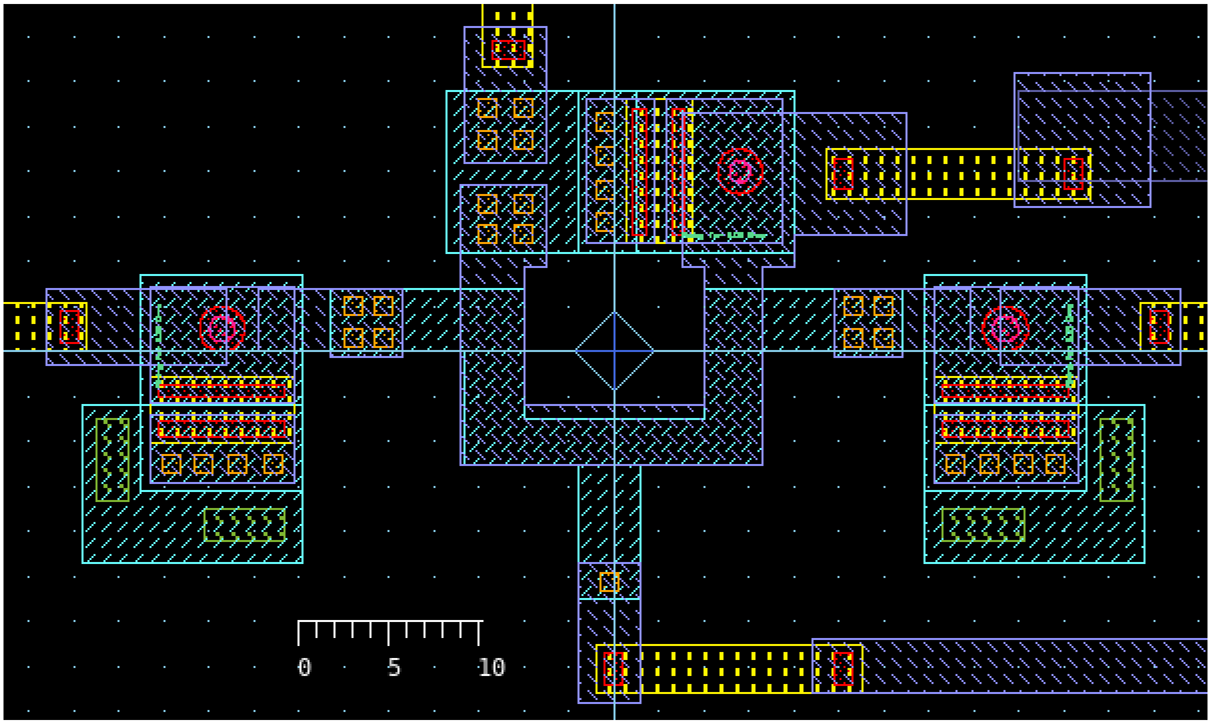


Figure S2. (color online) Schmatic layout of the synaptic test circuit. The Josephson junctions are the three red circles. The two on the bottom compose the SQUID neuron and the one on the top is a 100 uA synaptic junction. The five larger yellow rectangles are resistors that isolate the circuit from the incoming wiring used to test the circuit. The three smaller yellow rectangles are shunt resistors to the ground plane. The center “U” shape is the inductive coupling from the synaptic JJ to the SQUID neuron. A ground plan is present under circuit, but is not shown for clarity. The scale bar on the lower left side represents 10 μm.

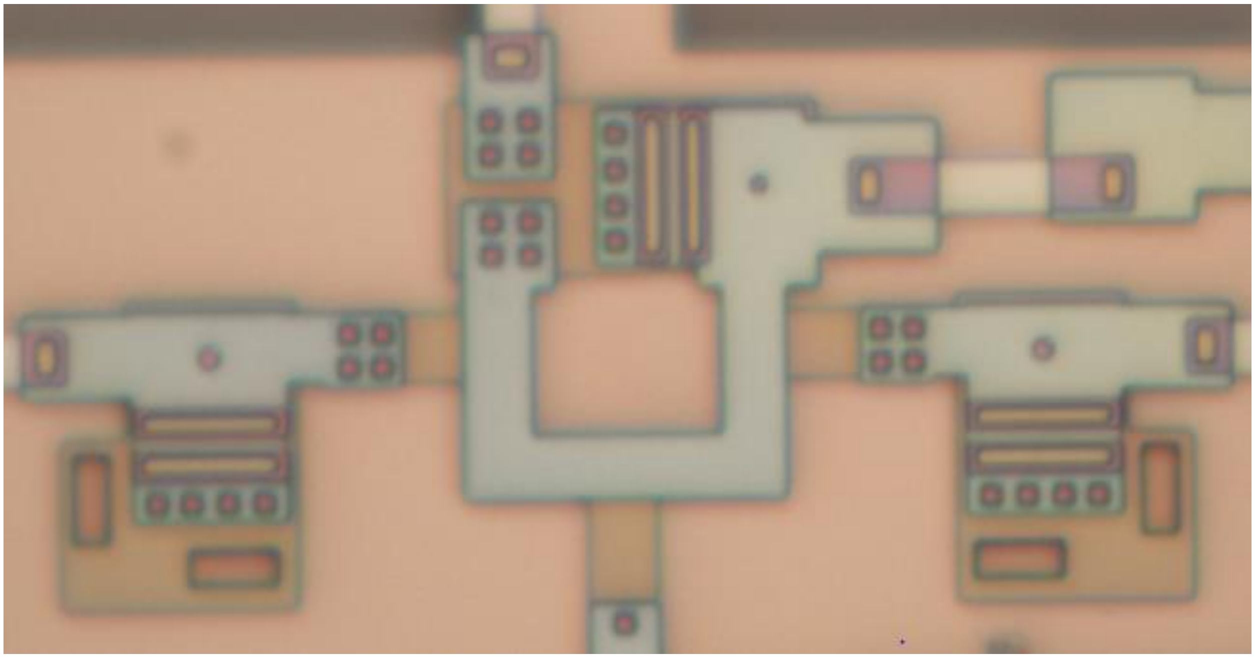

Figure S3. (color online) optical image of the JJ synapse and SQUID neuron shown schematically in figure S2.

## References:


1 McCumber, D. Effect of ac impedance on dc voltage-current characteristics of superconductor weak-link junctions. *Journal of Applied Physics* **39**, 3113-3118 (1968).

2 Stewart, W. Current-voltage characteristics of Josephson junctions. *Applied Physics Letters* **12**, 277-280 (1968).
